# Classification of First-Episode Schizophrenia Patients and Healthy Subjects by Automated MRI Measures of Regional Brain Volume and Cortical Thickness

**DOI:** 10.1371/journal.pone.0021047

**Published:** 2011-06-21

**Authors:** Yoichiro Takayanagi, Tsutomu Takahashi, Lina Orikabe, Yuriko Mozue, Yasuhiro Kawasaki, Kazue Nakamura, Yoko Sato, Masanari Itokawa, Hidenori Yamasue, Kiyoto Kasai, Masayoshi Kurachi, Yuji Okazaki, Michio Suzuki

**Affiliations:** 1 Department of Neuropsychiatry, University of Toyama, Toyama, Japan; 2 Department of Psychiatry, Tokyo Metropolitan Matsuzawa Hospital, Tokyo, Japan; 3 Department of Neuropsychiatry, Graduate School of Medicine, University of Tokyo, Tokyo, Japan; 4 Tosa Hospital, Kochi, Japan; 5 Department of Radiology, Tokyo Metropolitan Matsuzawa Hospital, Tokyo, Japan; 6 Tokyo Institute of Psychiatry, Tokyo, Japan; The University of Melbourne, Australia

## Abstract

**Background:**

Although structural magnetic resonance imaging (MRI) studies have repeatedly demonstrated regional brain structural abnormalities in patients with schizophrenia, relatively few MRI-based studies have attempted to distinguish between patients with first-episode schizophrenia and healthy controls.

**Method:**

Three-dimensional MR images were acquired from 52 (29 males, 23 females) first-episode schizophrenia patients and 40 (22 males, 18 females) healthy subjects. Multiple brain measures (regional brain volume and cortical thickness) were calculated by a fully automated procedure and were used for group comparison and classification by linear discriminant function analysis.

**Results:**

Schizophrenia patients showed gray matter volume reductions and cortical thinning in various brain regions predominantly in prefrontal and temporal cortices compared with controls. The classifiers obtained from 66 subjects of the first group successfully assigned 26 subjects of the second group with accuracy above 80%.

**Conclusion:**

Our results showed that combinations of automated brain measures successfully differentiated first-episode schizophrenia patients from healthy controls. Such neuroimaging approaches may provide objective biological information adjunct to clinical diagnosis of early schizophrenia.

## Introduction

Schizophrenia is a disabling psychiatric disorder which usually begins to affect individuals during their adolescence or early adulthood and most patients continue to suffer social, economic, and psychological difficulties from the first manifestation of the illness. Currently, diagnoses of psychiatric disorders are made on the basis of clinical manifestations and associated psycho-social disturbances [1], [2]. However, there is an evidence for diagnostic instability in psychotic patients at an early stage of illness [3], [4]. Although an accurate diagnosis is considered a prerequisite for appropriate physical/psychological treatment for each patient, no objective biomarker has been identified.

Previous structural magnetic resonance imaging (MRI) studies have demonstrated gray matter reductions of fronto-temporolimbic brain regions in schizophrenia patients compared with those of healthy subjects [5]–[11]. Several MRI-based studies have attempted to distinguish schizophrenia patients from healthy subjects using a variety of approaches such as manually traced regions of interest (ROI) [12], [13], voxel-based morphometry (VBM) [14]–[16], cortical pattern matching [17], and cortical thickness obtained by a surface-based approach [18]. These studies have generally reported high classification accuracies (ranging from 75% to 92%), suggesting the potential clinical (i.e., diagnostic) utility of structural MRI. The majority of such classification studies employed chronic schizophrenia patients [12], [14]–[16], [18]. To date, only two studies [13], [17] have attempted to distinguish between first-episode patients and healthy subjects by structural MRI.

Recently, an automated surface-based approach which can reliably measure local mean cortical thickness has been developed [19]. Several MRI studies applying this technique to schizophrenia have yielded robust findings such as cortical thinning especially in prefrontal and temporal regions [20]–[25]. This surface-based approach also enables to perform cortical parcellation and measurement of regional cortical volumes [25]–[27]. These approaches have been validated by several studies [21], [26], [28], [29]. By using these newly developed automated methods to assess brain morphology (i.e., cortical thickness and regional brain volumes). Desikan et al. [30] demonstrated successful classification of subjects with mild cognitive impairment, patients with Alzheimer's disease, and controls. To our knowledge, however, no studies have attempted to classify patients with schizophrenia and healthy subjects with this fully automated MRI-based analysis.

In this study, we intended to classify schizophrenia patients and healthy subjects using discriminant analysis with automated MRI-based measures of regional brain volume and cortical thickness. On the basis of findings of previous studies, we hypothesized that (1) cortical thinning and gray matter volume reductions in prefrontal and temporal regions would be seen in schizophrenia patients compared with controls, (2) and these MRI measures would differentiate schizophrenia patients from healthy subjects with good accuracy.

## Materials and Methods

### Subjects

Fifty-two patients (29 males, 23 females) with first-episode schizophrenia were recruited from the inpatient population at Tokyo Metropolitan Matsuzawa Hospital. Inclusion criteria for first-episode schizophrenia patients were (1) first psychiatric hospitalization, (2) younger than 45 years old, (3) currently psychotic as reflected by the presence of at least one “positive” symptom, and (4) fulfilling the ICD-10 research criteria for schizophrenia. Two experienced psychiatrists separately examined the patients within two weeks of admission and diagnostic consensus was confirmed. Furthermore, thorough medical record review was performed to confirm the diagnostic stability for all the patients during the follow-up periods (1 to 5 years) after first admission. All but three male patients with schizophrenia were right-handed. All patients had received antipsychotic medications at the time of scanning.

The control subjects consisted of 40 healthy volunteers (22 males, 18 females) who were recruited from the hospital staff and college students. All of the control subjects were right-handed. All control subjects were interviewed by psychiatrists using the questionnaire concerning their family and past histories, and present illness. Individuals who had a personal history of psychiatric illness or a family history of psychiatric disorders in their first degree relatives were excluded.

For the discriminant analysis described below, the subjects were randomly assigned to two independent groups. The first group consisted of 36 males (16 healthy subjects and 20 schizophrenia patients) and 30 females (13 healthy subjects and 17 schizophrenia patients). The second group for the prospective validation consisted of 15 males (6 healthy subjects and 9 schizophrenia patients) and 11 females (5 healthy subjects and 6 schizophrenia patients). Since the sample size of the present study is relatively modest, we assigned more subjects to the first group (i.e., about 70%) than to the second group to enhance the discriminating ability of the classifier.

In the schizophrenia patients, clinical symptoms were assessed using the Brief Psychiatric Rating Scale (BPRS) [31]. The premorbid IQ for schizophrenia patients and the present IQ for control subjects were estimated using the shortened version of the Japanese version of the National Adult Reading Test (JART) [32]. The subjects' socio-economic status (SES) as well as parental SES was assessed using the Hollingshead's Index [33].

All subjects were physically healthy at the time of the study, and none had a lifetime history of serious head trauma, neurological illness, or serious medical or surgical illness. Individuals who met the ICD-10 research criteria for mental and behavioral disorders due to psychoactive substance use were excluded. All schizophrenia patients participated in this study after providing written informed consent. In addition, regal representatives of schizophrenia patients gave written informed consent. In case of unable to directly access to a patient's legal representative, oral informed consent was obtained using telephone, and this procedure was witnessed by at least two hospital staff and recorded in the medical chart. All control subjects also provided written informed consent. Since control group of this study consisted of only healthy adults, their legal representatives were not asked to give informed consents. This study was approved by the Committee on Medical Ethics of Tokyo Metropolitan Matsuzawa Hospital.

### MRI data acquisition

MR images were obtained using a Philips Intera 1.5-T scanner (Philips Medical Systems, Best, Netherlands) with a three-dimensional sequence yielding 192 contiguous T1-weighted slices of 1.0-mm thickness in the axial plane. The imaging parameters were as follows: repetition time = 21 ms, echo time = 9.2 ms, flip angle = 30°, field of view = 256 mm, matrix size = 256×256 pixels, voxel size = 1.0×1.0×1.0 mm^3^.

### Automated MRI data processing

Cortical reconstruction and volumetric segmentation were performed with the Freesurfer image analysis suite (version 4.5), which is documented and freely available for download online (http://surfer.nmr.mgh.harvard.edu/). This processing includes motion correction and averaging of multiple volumetric T1-weighted images, removal of non-brain tissue using a hybrid watershed/surface deformation procedure [34], automated Talairach transformation, segmentation of the subcortical white matter and deep gray matter volumetric structures (including hippocampus and amygdala) [35], [36], intensity normalization [37], tessellation of the gray matter/white matter boundary, automated topology correction [38], [39], and surface deformation following intensity gradients to optimally place the gray/white and gray/cerebrospinal fluid (CSF) borders at the location where the greatest shift in intensity defines the transition to the other tissue class [19], [40], [41]. Once the cortical models are completed, a number of deformable procedures can be performed for further data processing and analysis.

**Figure 1:**
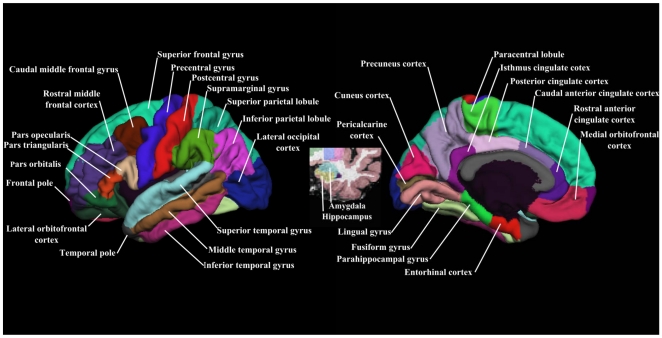
Representations of ROIs examined in this study on the left hemisphere. Cortical ROIs are shown in lateral view (left) and medial view (right). Two subcortical ROIs (i.e., amygdala and hippocampus) are visible in coronal view (middle).

Cortical thickness measurements were obtained by calculating the shortest distance from the gray/white boundary to the gray/CSF boundary at each vertex on the tessellated surface [19]. The cerebral cortex of each MRI scan was automatically parcelled into regions of interest (ROIs) based on gyral and sulcal structure [26], [42]. Both automated cortical thickness measurements and cortical parcellation have already been validated [21], [26], [28], [29]. Figure 1 presents the neocortical ROIs and two limbic ROIs (hippocampus and amygdala) examined in this study. To control for head size in statistical analyses, the total intracranial volume (ICV) was calculated automatically [43].

### Statistical analysis-1 Group comparison

Demographic and clinical variables were compared by analysis of variance (ANOVA). The ROI volumes and the mean cortical thickness of ROIs were analyzed by repeated measures analysis of covariance (ANCOVA) with diagnosis and gender as between-subject factors, hemisphere (left, right) as a within-subject factor, and age and ICV as covariates. To prevent possible type 1 error, we used false positive discovery rate (FDR) correction. For variables of which p-values remained significant even after the FDR correction, post hoc Scheffe's tests were used to follow up significant main effects or interactions.

### Statistical analysis-2 Classification by brain measures

The following statistical procedures were carried out separately for each gender, as was the case in our previous studies [12], [13], on the basis of the gender differences in brain morphology found in this study (described below) as well as the evidence for gender differences in brain morphology among healthy subjects [44] and gender-specific brain structural changes in schizophrenia patients [45], [46].

#### Transformation of brain measures into z scores

The volumes and mean cortical thickness of ROIs were expressed as standardized z scores corrected by regression analysis for the variations in head size and age of the control subjects, as described in our previous studies [12], [13]. Briefly, the ROI volume and mean cortical thickness for the control group were regressed against ICV and age, yielding a residual value for each control subject. The ROI volume and mean cortical thickness for the patient groups were entered into the same equation as for the control group to calculate the residual value for each patient. The mean residual values and standard deviation (SD) derived from the control subjects were used to calculate z scores (z = [residual value - mean residual value for control subjects]/SD). For the control subjects, the expected mean z score was 0 with an SD of 1. The use of standardized z scores allows analysis of disease-related changes independent of head size and normal aging.

#### Linear discriminant function analysis

For the first group, discriminant function analysis was conducted using z scores as independent variables to assess the possibility of classifying diagnostic groups by a combination of brain measures. The variables were entered in a stepwise manner. Since we employed a stepwise variable selection, the number of variables which were entered into the discriminant analysis varied depending on the inclusion and exclusion criteria. In this study, relatively conservative inclusion criteria were used for the stepwise selection, which were set at p<0.05 to enter and p>0.1 to remove. If we used a more liberal criterion, more variables could be used for the discriminant function, vice versa. For each step, always a measure whose p-value is the smallest and smaller than 0.05 is entered to the discriminant function. Similar to a stepwise linear regression analysis, however, p-values of variables vary for each step. If a p-value of a measure that has already been entered to the model exceeds 0.1, this variable is removed at this step. If a p-value of the measure is 0.06 (i.e., <0.1), it remains in the model. However, if a measure with a p-value of 0.06 has yet to be entered in the model, it is still out of the model at this step. For each subject of the second group, the discriminant score was calculated using the discriminant function derived from the first group and his/her diagnosis was predicted based on the discriminant score. Since the p-value for the stepwise variable selection was computed solely from the first group, the classification of the second group was achieved independently of subjects' diagnosis of the second group. Sensitivity, specificity, accuracy, positive predictive value (PPV), negative predictive value (NPV), and false positive rate (FPR) of the classifier were calculated. Detailed descriptions of discriminant function analysis and stepwise variable selection can be found at the Statsoft website (http://www.statsoft.com/textbook/).

All statistical analyses were performed using the STATISTICA 06J software package (Statsoft, Tulsa, OK).

## Results

### Demographic and clinical characteristics

Tables 1 and 2 present the results of group comparison of the demographic and clinical measures of male subjects and female subjects, respectively. When all subjects were combined, there were significant main effects of diagnosis on SES (F = 41.77, df = 1,87, p<0.001) and estimated IQ (F = 6.90, df = 1,85, p = 0.01). Post hoc tests showed that schizophrenia patients had lower SES (p<0.001) and lower estimated IQ (p = 0.01) than controls.

**Table 1:** Demographic and clinical characteristics of the male subjects.

A. First group	Control subjects		Schizophrenia patients		Analysis of variance	
	n = 16		n = 20			
	Mean	SD	Mean	SD	F	p
Age (years)	29.9	5.6	27.8	6.0	1.19	0.28
Handedness (number of right-handed)	16.0		19.0			
Socio-economic status	1.6	0.5	2.7	1.0	13.07	0.001
Parental socio-economic status	2.3	0.6	2.4	0.8	0.23	0.63
Estimated IQ	108.8	7.9	103.0	9.7	3.68	0.06
Duration of illness (months)			9.9	11.1		
Total BPRS score			40.2	11.5		
Antipsychotic medication (mg/day, chlorpromazine equiv.)			1074.5	487.9		

B. Second group	Control subjects		Schizophrenia patients		Analysis of variance	
	n = 6		n = 9			
	Mean	SD	Mean	SD	F	p
Age (years)	30.8	6.0	27.9	6.8	0.74	0.41
Handedness (number of right-handed)	6.0		7.0			
Socio-economic status	1.8	0.5	3.9	1.6	9.68	0.01
Parental socio-economic status	2.3	0.4	2.3	0.7	0.37	0.56
Estimated IQ	111.7	5.1	106.2	10.6	1.38	0.26
Duration of illness (months)			12.5	13.0		
Total BPRS score			42.5	9.9		
Antipsychotic medication (mg/day, chlorpromazine equiv.)			864.4	637.7		

**Table 2:** Demographic and clinical characteristics of the female subjects.

A. First group	Control subjects		Schizophrenia patients		Analysis of variance	
	n = 13		n = 17			
	Mean	SD	Mean	SD	F	p
Age (years)	27.5	4.8	28.1	5.8	0.16	0.69
Handedness (number of right-handed)	14.0		17.0			
Socio-economic status	1.6	0.5	3.1	1.1	17.14	<0.001
Parental socio-economic status	2.4	0.8	2.9	0.8	3.03	0.09
Estimated IQ	107.0	8.1	103.5	7.8	2.55	0.12
Duration of illness (months)			13.0	12.6		
Total BPRS score			37.4	9.7		
Antipsychotic medication (mg/day, chlorpromazine equiv.)			930.8	451.6		

B. Second group	Control subjects		Schizophrenia patients		Analysis of variance	
	n = 5		n = 6			
	Mean	SD	Mean	SD	F	p
Age (years)	28.4	3.8	28.3	8.6	0.05	0.83
Handedness (number of right-handed)	4.0		6.0			
Socio-economic status	1.6	0.5	2.5	1.0	4.65	0.06
Parental socio-economic status	2.0	0.0	2.6	0.9	2.25	0.17
Estimated IQ	108.1	10.8	103.0	8.1	0.18	0.68
Duration of illness (months)			14.5	19.8		
Total BPRS score			36.5	4.4		
Antipsychotic medication (mg/day, chlorpromazine equiv.)			483.3	263.9		

### Comparison of the brain measures

Tables S1 and S2 show the comparisons of the volumes and the mean cortical thicknesses of ROIs among diagnostic groups, respectively. Below, we describe the significant results of post hoc tests.

#### Comparison of the ROI volumes

**Figure 2:**
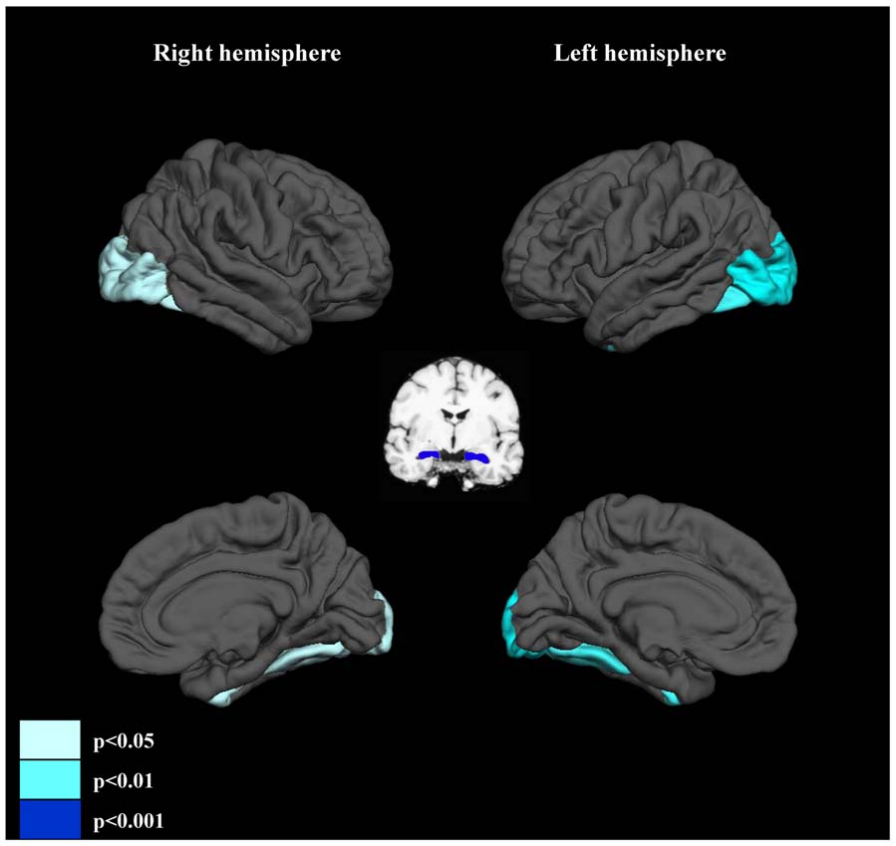
ROIs for which the volumes were significantly reduced in schizophrenia patients compared with those of healthy subjects. ROIs were differentially colored according to the p values of the post hoc tests.

Post hoc tests demonstrated significant gray matter volume reductions of the bilateral hippocampus (p<0.001 for both hemispheres), the bilateral fusiform gyri (p = 0.002 for left, p = 0.024 for right), and the bilateral lateral occipital cortices (p = 0.001 for left, p = 0.014 for right) in schizophrenia patients compared with those of healthy subjects (Figure 2). Gender differences of ROI volumes were seen in the bilateral amygdala (male>female, p<0.001 for both hemispheres).

#### Comparison of the mean thickness of ROIs

**Figure 3:**
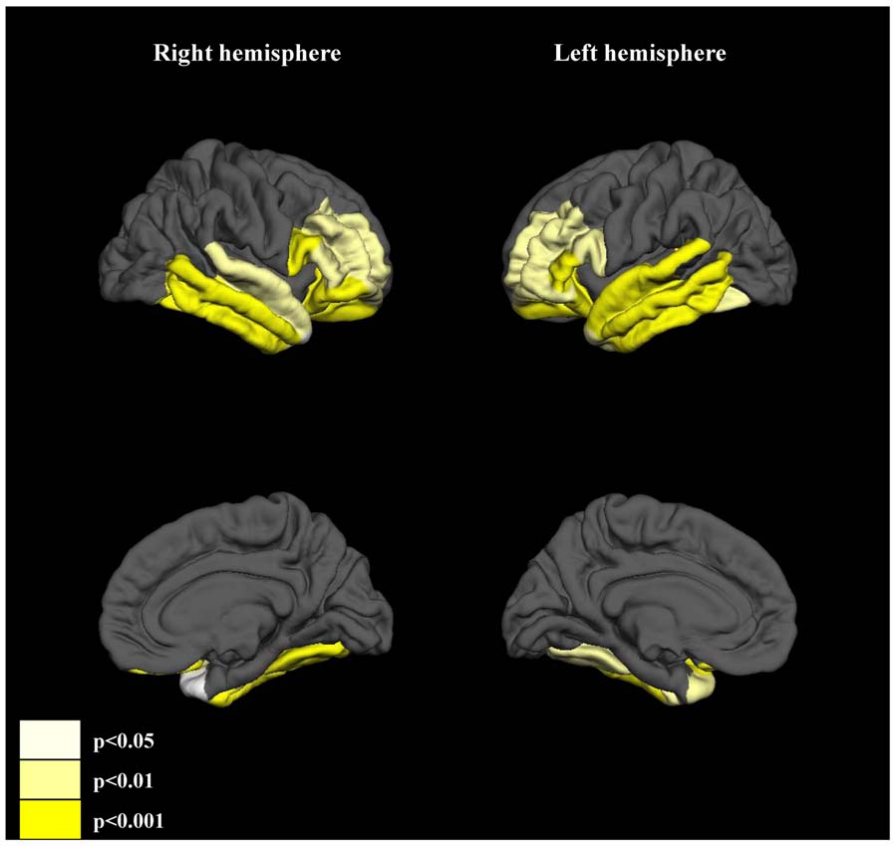
Significant cortical thinning of ROIs in schizophrenia patients compared with that of healthy subjects observed in this study. ROIs were differentially colored according to the p values of the post hoc tests.

Significant cortical thinning in schizophrenia patients compared with controls was observed in the bilateral rostral middle frontal gyri (p = 0.007 for left, p = 0.007 for right), the bilateral pars opecularis (p = 0.002 for left, p<0.001 for right), the bilateral pars triangularis (p<0.001 for left, p = 0.009 for right), the bilateral pars orbitalis (p = 0.002 for left, p<0.001 for right), the bilateral lateral orbitofrontal cortices (p<0.001 for both hemispheres), the bilateral superior temporal gyri (p<0.001 for left, p = 0.001 for right), the bilateral middle temporal gyri (p<0.001 for both hemispheres), the bilateral inferior temporal gyri (p<0.001 for both hemispheres), the bilateral fusiform gyri (p = 0.005 for left, p<0.001 for right), and the bilateral temporal pole (p = 0.004 for left, p = 0.04 for right) (Figure 3).

### Classification of schizophrenia patients and healthy subjects by brain measures

**Figure 4:**
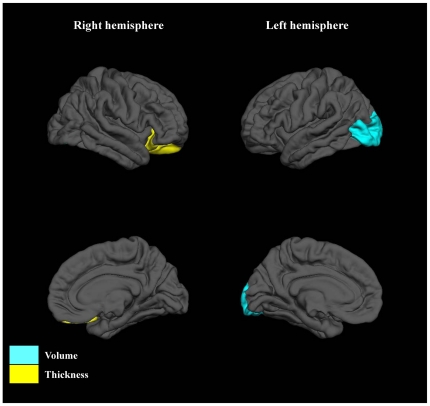
Discriminative pattern for male subjects. Selected regions were differentially colored when volume (blue) or thickness (yellow) of those regions were entered into the model.

Among male subjects, the following 2 measures were entered in a stepwise manner: the left lateral occipital cortex volume and right lateral orbitofrontal cortex thickness (Figure 4). Accuracy, sensitivity, specificity, PPV, NPV and FPV of the obtained classifier were 86.1%, 80.0%, 93.8%, 94.1%, 78.9%, and 5.9%, respectively in the first male cohort. In the second cohort, the classifier correctly assigned 86.7% of the subjects. Accuracy, sensitivity, specificity, PPV, NPV and FPV for second cohort were 86.7%, 88.9%, 83.3%, 88.9%, 83.3%, and 11.1%, respectively (Table 3).

**Table 3:** Classification performance.

A. First group	Male (n = 36)		Female (n = 30)	
	Predicted diagnosis		Predicted diagnosis	
	HC	SZ	HC	SZ
Clinical diagnosis				
HC	15	1	13	0
SZ	4	16	1	16
Accuracy (%)	86.1		96.7	
Sensitivity (%)	80.0		94.1	
Specificity (%)	93.8		100.0	
PPV (%)	94.1		100.0	
NPV (%)	78.9		92.9	
FPR (%)	5.9		0.0	

B. Second group	Male (n = 15)		Female (n = 11)	
	Predicted diagnosis		Predicted diagnosis	
	HC	SZ	HC	SZ
Clinical diagnosis				
HC	5	1	5	0
SZ	1	8	2	4
Accuracy (%)	86.7		81.2	
Sensitivity (%)	88.9		66.7	
Specificity (%)	83.3		100.0	
PPV (%)	88.9		100.0	
NPV (%)	83.3		71.4	
FPR (%)	11.1		0.0	

FPR, false positive rate; HC, healthy control; NPV, negative predictive value; PPV, Positive predictive value; SZ, schizophrenia.

**Figure 5:**
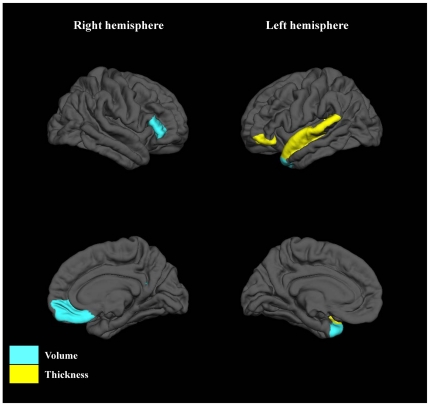
Discriminative pattern for female subjects. Selected regions were differentially colored when volume (blue) or thickness (yellow) of those regions were entered into the model.

During the stepwise procedure, the following 5 measures were selected as variables in female subjects: the left temporal pole volume, the right medial orbitofrontal cortex volume, the right pars triangularis volume, the left pars orbitalis thickness, and the left superior temporal gyrus thickness (Figure 5). Accuracy, sensitivity, specificity, PPV, NPV and FPV of the classifier were 96.7%, 94.1%, 100%, 100%, 92.9%, and 0%, respectively in the first female cohort. Obtained classifier correctly classified 81.2% of the subjects of the second cohort. Accuracy, sensitivity, specificity, PPV, NPV and FPV for the second cohort were 81.2%, 66.7%, 100%, 100%, 71.4%, and 0%, respectively (Table 3).

## Discussion

### Classification performance

To the best of our knowledge, this is the first MRI study to reliably classify first-episode patients with schizophrenia and healthy subjects using fully automated MRI-based discriminant analysis based on both brain regional volumes and regional cortical thicknesses. Our results were comparable to those of previous MRI-based classification studies in chronic [14]–[16], [18] and first-episode [13], [17] schizophrenia patients. Our results suggest that the combination of automated brain measures is a candidate for an objective biological marker of early schizophrenia adjunct to clinical diagnosis.

In the present study, the fronto-temporolimbic regions as well as the occipital cortex exhibited the discriminative patterns among the diagnostic groups. These patterns appear to be somewhat different from those of previous classification studies between schizophrenia patients and healthy controls using whole brain analysis by VBM [16] or cortical pattern matching [17], which highlighted the fronto-temporal regions as contributing to between-group differentiation. Interestingly, we replicated recent findings by Rimol et al. [47] in showing robust cortical thinning of posterior cortices in first-episode schizophrenia. Our results might thus suggest that combination of cortical thickness (including occipital regions) and gray matter volume contributed to high classification accuracies reported in this study.

Several studies have attempted to distinguish between persons with psychiatric conditions and healthy controls using neuropsychological tests [48], a combination of structural brain measures and neuropsychological tests [49], and functional MRI [50]. Although these previous studies also reported high classification accuracy, neuropsychological and functional measures are considered more susceptible to the subjects' condition (i.e., state-dependent). In contrast, brain morphologic changes in schizophrenia are considered to be more static and already exist at the first episode of the illness [5] or even before/during the onset of overt psychosis [51]–[53] Our findings that MRI measures alone could reliably differentiate healthy controls and schizophrenia patients might thus suggest a role of brain structural measures in the earlier detection of psychosis. In fact, a recent VBM-based classification study demonstrated successful discrimination of individuals with at risk mental state (ARMS) who later developed psychosis from those without transition to psychosis [54].

### Volume reductions and cortical thinning of ROIs in patients

This study demonstrated significant gray matter volume reductions of temporal, limbic, and occipital regions in schizophrenia patients compared with those of controls. In schizophrenia patients, significant cortical thinning was more widely observed, relative to volume reductions, in prefrontal and temporal regions. These results are consistent with previous studies that reported fronto-temporolimbic gray matter volume reductions [5]–[11] and cortical thinning of prefrontal/temporal regions [20]–[25] in schizophrenia patients. Prefrontal and temporolimbic regions are considered to be involved in cognitive function, auditory/visual processing, speech, emotional processing, executive function, and decision-making, all of which are often impaired in schizophrenia patients [55]–[57]. Onitsuka et al. [58] demonstrated volume reductions of the bilateral occipital sub-region (the visual association areas), which largely includes the lateral occipital cortex where the schizophrenia patients had a decreased volume in this study. In general, the present study has replicated the brain structural abnormalities in schizophrenia patients demonstrated in previous MRI-based studies.

Gender difference was seen in the bilateral amygdala volume (male>female) in accordance with previous studies [44]. In order to exclude such gender effect which potentially confounds classification analyses, we divided the subjects into male and female cohorts in this study.

### Limitations

A few limitations in this study should be taken into account. First, this study was partly limited by the lack of inclusion of other psychiatric disorders such as bipolar affective disorder (BD). Our preliminary classification analysis using the current sample as well as 15 BD patients [8 males (mean age, 33.5 years) and 7 females (mean age, 33.7 years)] correctly assigned 81.4% of male subjects and 87.5% of female subjects, respectively (unpublished data). However, larger number of BD patients will be needed to delineate the conclusion that such technique may possibly attribute to the clinical diagnosis of different psychiatric conditions. Second, the higher socio-economic status of control group compared to schizophrenia patients might have confounded the analyses, although parental socio-economic status was not different between groups. Third, the results may have been influenced by antipsychotic medication that all patients in this study had received prior to scanning [59]–[61]. Finally, as the sample size of this study is modest (51 males and 41 females), we needed to assign more subjects to the training cohort than to the validation cohort in order to obtain more reliable classifiers. A larger number of subjects should be tested for validation in future study.

### Conclusion

In conclusion, our results showed that combinations of fully automated brain measures successfully classified diagnostic groups (i.e., schizophrenia patients and controls), and suggest that such neuroimaging approaches may provide objective biological information adjunct to clinical diagnosis of early schizophrenia.

## Supporting Information

Table S1 The results of comparison of the ROI volumes between schizophrenia patients and healthy controls.
(XLSX)

Table S2 The results of comparison of the mean thickness of the ROIs between schizophrenia patients and healthy controls.
(XLSX)
